# Pharmacological effects of medicinal components of *Atractylodes lancea* (Thunb.) DC.

**DOI:** 10.1186/s13020-018-0216-7

**Published:** 2018-11-27

**Authors:** Xie Jun, Peng Fu, Yu Lei, Peng Cheng

**Affiliations:** 1 Chengdu University of Traditional Chinese Medicine, Chengdu, China; 2 West China School of Pharmacy, Sichuan University, Chengdu, China

**Keywords:** Natural product, AL, Pharmacological activities

## Abstract

*Atractylodes lancea* Thunb. DC. (AL) has a long history as one of the important herbs used in East Asia. This review is on the purpose of providing a comprehensive summary of the pharmacological effects of AL and its extractions. The publication from PubMed, ScienceDirect, Springer, and Wiley database was collected and summarized. The potential application of AL on the disease could be attributed to its pharmacological properties such as anti-cancer, anti-inflammatory and other essential effects. Hence, this review aims at providing evidence of the pharmacological activities of AL as one of natural products used in clinical trial.

## Background

**Fig. 1:**
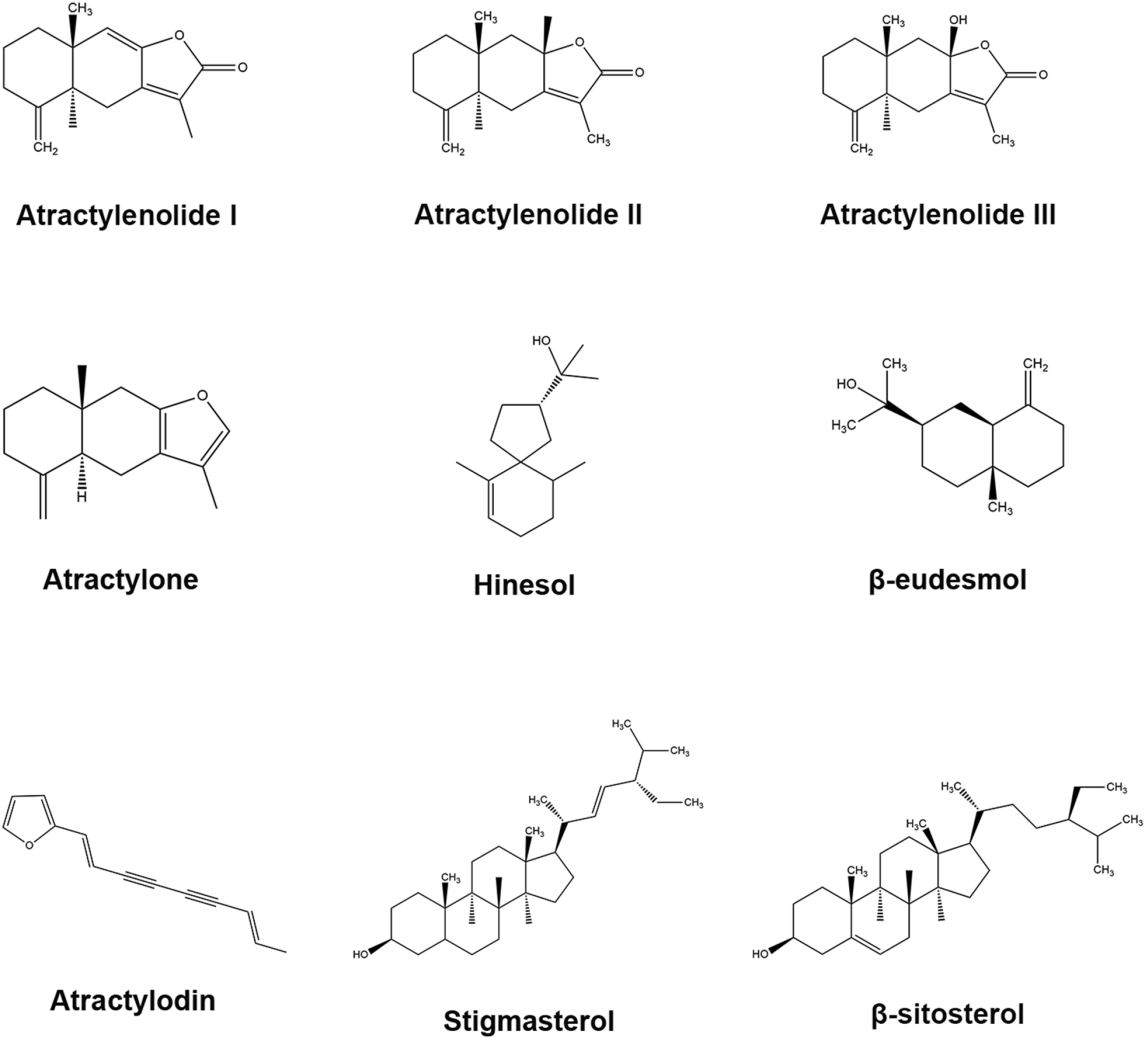
The chemical structures of pure compounds extracted from AL (drawing via ChemDraw)

*Atractylodes lancea* Thunb. DC. (AL) has a long history as one of the important herbs used in East Asia. AL is grown mainly in Hubei and Jiangsu. In traditional Chinese medicine (TCM), the rhizome of AL tastes pungent and bitter, and belongs to spleen, stomach and liver meridian. AL could eliminate dampness, strengthen the spleen, eliminating wind and dispersing cold. According to TCM theory, AL was traditionally used to treat rheumatic diseases, digestive disorders, night blindness, and influenza [1]. AL was generally applied into traditional decoction, such as Ermiao Powder, Simiao Powder, Yueju-Wan and several other famous decoctions. Recently, it was interesting to find the extract from AL could also exert anti-cancer, anti-obesity and anti-inflammatory effects [2]. AL contains sesquiterpenes, sesquiterpenoids, polyethylene alkynes, phytosterols and etc., such as elemol, β-selinene and atractylone [3, 4]. Sesquiterpenes are a class of natural products composed of three isoprene units, derived from the 15-carbon farnesyl pyrophosphate. Sesquiterpenoids are extensively distributed in nature with 15-carbon after biochemical modifications of sesquiterpenes. Alkynes are a class of organic compounds composed of at least one carbon–carbon triple bond, easily undergoing oxidative cleavage. Phytosterols are a subgroup of the steroids. Steroids are a class of organic molecules composed of seventeen carbon atoms, with four rings arranged in a specific molecular configuration. Especially, this review article will concentrate on the major constituents identified in AL with multi-pharmacological activities [5, 6]. The names are shown in Table 1 and structures are shown in Fig. 1.

**Table 1:** The chemical information of major components of AL

Chemical name	Formula	Type of compound	Molecular weight (g/mol)
Atractylenolide I	C_15_H_18_O_2_	Sesquiterpene	230.30
AtractylenolideII	C_15_H_20_O_2_	Sesquiterpene	232.32
Atractylenolide III	C_15_H_20_O_3_	Sesquiterpene	248.32
Atractylone	C_15_H_20_O	Sesquiterpenoid	216.32
Hinesol	C_15_H_26_O	Sesquiterpenoid	216.3
β-Eudesmol	C_15_H_26_O	Sesquiterpenoid	222.37
Atractylodin	C_13_H_10_O	Polyethylene alkyne	182.22
Stigmasterol	C_29_H_48_O	Phytosterol	412.69
β-Sitosterol	C_29_H_50_O	Phytosterol	414.71

## Pharmacological effects

### *Atractylodes lancea* Thunb. DC.

Opisthorchis viverrini (OV)-induced cholangiocarcinoma (CCA) is the common cancer between male and female, especially in Thailand. Recent study exhibited that the ethanol extract of AL exerted an inhibitory effect on OV-induced CCA animal model without obvious toxicity through oral administration for 30 days according to the results from positron emission tomography-computed tomography and male Syrian hamsters [7]. In order to explore the effect of AL rhizome on gastrointestinal motility, the atropine-, dopamine-, and 5-hydroxytryptamine (5-HT)-treated mice were adopted in the study. The results exerted that 1 mg/kg extract could vitally suppress dopamine-induced gastric emptying and gastrointestinal motility decrease through intraperitoneal injection. The extract at the concentration of 1000 mg/kg could restrict atropine-induced small intestinal motility decrease, and the extract of 500 mg/kg dose could block 5-HT-induced gastric emptying and small intestinal motility decrease [8]. Additionally, AL exerted anti-gastric ulcer effect in the rat model through decreasing inflammatory mediators (i.e. tumor necrosis factor-α (TNF-α), interleukin-8 (IL-8), IL-6), and prostaglandin E_2_ (PGE_2_) and increasing epidermal growth factor (EGF) and trefoil factor2 (TFF2) [9]. Soil-borne fungal pathogens reduced 30% yields of peanuts [10]. Recently, it was reported that intercropping with the peanuts, AL could also suppress soil-borne fungal diseases through the inhibition of *Fusarium oxysporum* growth [11]. Petroleum ether (PE) extraction from AL showed an inhibitory effect on BGC-823 and SGC-7901 human gastric cancer cells. PE fraction could induce apoptosis, cell cycle arrest, and mitochondrial transmembrane potential changes [12]. A recent report exerted that MeOH extract of AL could inhibit nitric oxide (NO) production in lipopolysaccharide-stimulated murine macrophage-like RAW264.7 cells and induce granulocyte-colony stimulating factor (G-CSF) secretion in murine normal colonic epithelial MCE301 cells. NO is a free radical [13]. The ethanol extract of AL exerted an essential inhibitory effect on lipase with the IC_50_ 9.06 µg/mL in a human pancreatic lipase assay, and exhibited anti-obesity effect on a high-fat diet-induced obesity mice model at the high concentration of 500 mg/kg [14]. The water extracts from AL showed the antagonistic activity against serotonin receptors and an inhibitory effect on the selective 5-HT2A/2C agonist 1-(2,5-dimethoxy-4-iodophenyl)-2-aminopropane-induced head-twitch response in mice [15]. Water extracts from AL reduced triptolide-induced toxicity through the down-regulation of the hepatic expression of CYP3A. In the meantime, AL could improve anti-inflammatory effects of triptolide. These findings suggested that AL could inhibit toxicity and increase therapeutic activity of triptolide through combination [16].

### Atractylenolide I

Atractylenolide I (ATL-I), a sesquiterpene, can be extracted from *Rhizoma Atractylodis Macrocephalae* (RAM) and AL [17]. ATL-I is soluble in Ethanol and Methanol, and is stable without exposure to light or moisture. ATL-I exerted multiple therapeutic activities, such as anti-cancer, anti-inflammatory and anti-atopic effects [18–20]. ATL-I was identified in AL as one of the main components in 2009 [21]. Bladder cancer ranks the second most common urological worldwide [22]. ATL-I recently was demonstrated to have the anti-tumor effect on bladder cancer in vivo and in vitro. It was found that ATL-I increased the p21 level and decreased the level of cyclin B1, CDK1 and Cdc25c, resulting in the inhibition of bladder cancer cell proliferation. Further study showed that ATL-I could induce cell cycle arrest in G2/M phase. Additionally, ATL-I had a stimulatory effect on apoptosis through the suppression of PI3 K/Akt/mTOR signaling pathway. The animal study showed that ATL-I could inhibit tumor growth without the obvious toxicity [17]. Leukemia refers to a type of cancers generally starting from the bone marrow and resulting in abnormal white blood cells. Recent studies showed that ATL-I could inhibit AML and CML leukemia cell proliferation and growth. MTT assay showed that ATL-I could inhibit K562 CML and U937 AML. ELISA and IF assay exhibited that ATL-I could induce apoptosis. Additionally, ATL-I also promoted caspase-3 and caspase-9 activation through the cleavage of pro-caspase-3 and pro-caspase-9. Caspase-3 and caspase-9 are an enzymes initiating apoptotic pathway. Also, ATL-I could increase the CD14 and CD68 markers, leading to the cell differentiation of ATL-I-treated leukemia cells [23]. Melanoma belongs to the skin cancer [24]. Recent researches showed that ATL-I could inhibit human melanoma cells growth through the induction of apoptosis. Further study exerted that ATL-I could suppress melanoma cell migration through the inhibition on p-JAK2, p-STAT3, MMP-2 and MMP-9. MMP-2 and MMP-9 are matrix metallopeptidases involved in invasion and metastasis in multiple cancers. Interestingly, the overexpression of STAT3 would at least partly reverse the anti-cancer effect of ATL-I on melanoma cells [25]. The clinical study about the evaluation of therapeutic effect of ATL-I on gastric cancer cachexia patients has been conducted in recent years. According to the results about appetite, body weight, mid-arm muscle circumference, Karnofsky performance status (KPS) status, ATL-I could improve appetite and KPS status. Meanwhile, the IHC staining results showed that ATL-I could increase TNF-α expression and reduce IL-1 and proteolysis-inducing factor (PIF) production [26]. Intestinal epithelial (IEC-6) cells showed an important role in gastrointestinal disease with the ability to promote and accelerate the healing of mucosal ulcers and wounds. Recent study found that ATL-I could promote IEC-6 cell migration and proliferation, with an increase of cytosolic free Ca^2+^ concentration (Ca^2+^). Further study exerted that ATL-I could increase TRPC1 and PLC-_γ1_ in IEC-6 cells [27]. Moreover, ATL-I suppressed vascular smooth muscle cell proliferation, migration, lipid peroxidation and inflammatory responses induced by oxidized modified low-density lipoprotein [28]. A present research indicated that ATL-I had a protective effect on the acute lung injury in the lipopolysaccharide (LPS)-induced mice. The detailed mechanism was associated with suppression of toll like receptor 4 (TLR4) expression and NF-κB activation. NF-κB could promote the transcription of oncogenic genes, cytokine production and cell survival after inducers stimulation, such as ROS, TNFα and IL-1β. It was found that ATL-I powerfully suppressed lung wet-to-dry weight ratio. Simultaneously, attenuating the LPS-induced pathological changes in lung tissues included inflammatory cells infiltration, interalveolar septal thickening, and edema. The further study showed that treatment with ATL-I considerably down-regulated the release of TNF-α, IL-6, IL-1β, and IL-13, which were detected by ELISA. However, ATL-I could enhance the expression of IL-10 in BALF. LPS could induce conformational changes in the TLR4 receptors to activate TLR4. TLR4 activation would promote NF-κB expression and inflammatory cytokine production. Additionally, ATL-I also had an inhibitory effect on the LPS-induced TLR4 expression and NF-κB activation, which were measured by western blot analysis [29]. It was interesting that recent study showed that ATL-I could inhibit uridine 5′-iphospho-glucuronosyltransferases (UGTs) isoforms, especially UGT2B7 [30]. Additionally, ATL-I could inhibit 5-LOX production to decrease allergic response [31].

### Atractylenolide II

Atractylenolide II (ATL-II), a sesquiterpene, can be extracted from RAM and AL, exerting anti-cancer and anti-inflammatory activities. The melting point of ATL-II is 383 °C. ATL-II was identified in AL in 1998 [32]. Recent study showed that suppression of STAT3 signaling had a promoting effect on the anti-melanoma action of AT-II in B16 and A375 cells. 48 h AT-II treatment showed a dose-dependent manner inhibitory effect on p-STAT3, p-Src, and Mcl-1 and Bcl-xL in B16 and A375 cells. However, the suppressive effect of AT-II would be mostly reversed after the restoration of active variant of STAT3. The animal studies showed that 14-day administration of AT-II could obviously suppress tumor growth and inhibit STAT3 activation [33]. A present study manifested that AT-II could significantly inhibit human gastric carcinoma cell HGC-27 and AGS growth and migration in a dose-dependent manner. Further study showed that AT-II could suppress Bcl-2, p-Akt and p-ERK expression, and increase Bax expression [34].

### Atractylenolide III

Atractylenolide III (ATL-III), a sesquiterpene, is the major component in RAM, and could be extracted from AL. The melting point of ATL-III is 200–201 °C, and the freezing point of ATL-III is 392–394 °F. ATL-III showed the cytotoxic effect on cancer cells and the anti-inflammatory effect on macrophages [35, 36]. ATL-III was identified in AL in 2008 [37]. Mast cells could promote atopic dermatitis [38]. It was demonstrated that ATL-III possessed anti-tumor activity on TSLP-stimulated human mast cell, HMC-1 cells. The detailed mechanism was that ATL-III could inhibit the murine double minute 2 levels, mast cell proliferation, IL-13 and phosphorylated signal transducer and activator of transcription. In the mean time, ATL-III could promote p53 expression. Additionally, the release of pro-inflammatory cytokines stimulated by TSLP (IL-6, TNF-α, and IL-8) was reduced by ATL-III. Furthermore, the Bcl2 and pro-caspase-3 levels could be down regulated by ATL-III, whereas the caspase-3 activation and cleaved PARP levels could be up regulated by ATL-III [39]. Recent study showed that ATL-III exhibited anti-inflammatory effect on LPS-triggered RAW264.7 mouse macrophages. The detailed mechanism was associated with the inhibitory effect of ATL-III on the production of NO, PGE2, TNF-α and IL-6, and the suppressive effect of ATL-III on NF-κB and mitogen-activated protein kinase (MAPK) signaling pathways. ATL-III could dose-dependently inhibit NO, TNF-α, PGE2 and IL-6 release, and high concentration of ATL-III (100 μM) could suppress cytokines cyclooxygenase-2 (COX-2) expression via the inhibition on the activation of NF-κB and ERK1/2 [40]. Additionally, ATL-III exerted a neuroprotective effect in rats with learning and memory impairment through the inhibition of ROS production and protein kinase C levels [41].

### Atractylone

Atractylone was one of the main sesquiterpenic constituents of AL, displaying the anti-inflammatory action and anti-hepatotoxic effect [42–44]. The boiling point is 285.00–286.00 °C. Atractylone was identified in AL in 2006, reaching 9.35% [45]. Atractylone showed an inhibitory effect on allergic inflammation in an ovalbumin (OVA)-induced AR animal model. Further study exerted that Atractylone could also suppress caspase-1/NF-κB/MAPKs activation phorbol 12-myristate 13-acetate and calcium ionophore A23187 (PMACI)-induced human mast cell line (HMC-1) cells. In the animal model, Atractylone decreased rub scores, and reduced IL-1, IL-4, IL-5, IL-6, IL-13, COX-2, intercellular adhesion molecule-1, and macrophage inflammatory protein-2 expression [46]. Recently, atractylone could decrease influenza A virus (IAV)-induced pulmonary injury at the concentration of 40 mg/kg for 5 days in mice model. Further study showed that atractylone could inhibit IL-6, TNF-α and IL-1β, but promote Toll-like receptor 7 (TLR7), MyD88, tumor necrosis factor receptor-associated factor 6 interferon-β (IFN-β) expression [47]. It was reported that atractylone exerted a salutary effect on the mast cell-mediated allergic reactions. The results exhibited that atractylone could suppress compound 48/80-induced rat peritoneal mast cells (RPMCs) degranulation intracellular Ca^2+^ level ([Ca^2+^]), tryptase release, and histamine release. Additionally, atractylone could inhibit compound 48/80-induced p56lck tyrosine kinase activity in RPMCs. Also, atractylone showed an inhibitory effect on histidine decarboxylase activity and expression in PMACI-induced HMC-1 cells. Further study showed that atractylone could also inhibit tryptase and histamine releases in PMACI-induced HMC-1 cells. Atractylone could inhibit morphological alteration and filamentous actin formation in stem cell factor-stimulated RPMCs animal model [48].

### Hinesol

Hinesol is a sesquiterpenoid, which was 5–9% in AL. Hinesol exhibited the cytotoxic effect on cancer cells and a strong anti-inflammatory effect. Hinesol was clarified in AL in 2003 [49]. Hinesol could reduce nuclear fragmentation and DNA fragmentation, indicating that hinesol had an inhibitory effect on human leukemia HL-60 cells through apoptosis. The further study showed that hinesol could modulate c-Jun signaling pathway through the activation of c-Jun N-terminal kinase (JNK) [50]. A recent study indicated that hinesol could show the anti-gastric ulcer effect through a significant inhibitory effect on H^+^, K^+^-ATPase activity. The concentration of ATP and K^+^ could not modulate the inhibitory effect of hinesol, but the increase of Mg^2+^ concentration could promote the inhibitory effect of hinesol. Also, hinesol displayed a moderate inhibitory effect on Mg^2+^-ATPase and Ca^2+^-ATPase activity [51].

### β-Eudesmol

β-Eudesmol, a sesquiterpenoid alcohol, is the main constituent of AL. β-Eudesmol can be also extracted from *Teucrium ramosissimum* [52]. The melting point of β-eudesmol is 72–74 °C. Particularly, β-eudesmol demonstrated a strong inhibitory effect on cancer, and β-eudesmol could protect neuron from inflammatory damage. CCA, or bile duct cancer, which was an uncommon adenocarcinoma that originated from the epithelial cells of bile ducts, is becoming a vital public health problem worldwide. Recent study exerted that β-eudesmol could improve the anti-cancer effect of 5-fluorouracil and doxorubicin in human CCA KKU-100 cells with the high expression of NAD (P) H-quinone oxidoreductase 1 (NQO1). Also, β-eudesmol could inhibit cell growth, migration, NQO1 expression and activity in CCA [53]. β-Eudesmol showed an inhibitory effect on CCA growth and metastasis in CCA-xenografted nude mouse model according to the results from positron emission tomography-computed tomography (PET-CT). Also, β-eudesmol could prolong the survival time of CCA mice model [54]. Another study exerted that β-eudesmol could also induce apoptosis, cell cycle arrest at G1 phase, and the cleavage of caspase 3 and caspase 7 in CCA [55]. Primary liver cancer is the third-leading cause of cancer death all over the world [56]. Recent studies showed that β-eudesmol isomers could inhibit proliferation of human hepatocellular carcinoma Hep-G2 cells through the induction of apoptosis, according to haematoxylin–eosin and acridine orange ethidium bromide staining results. Further study showed that β-eudesmol could decrease mitochondrial membrane potential and activate caspases [57]. Recently, β-eudesmol showed the anti-tumor activities in human lung and colon cancer cells. Further study exerted that β-eudesmol (200 μM) significantly decreased A549 cells migration towards type IV collagen (54% inhibition) and fibronectin (60% inhibition). Meanwhile, β-eudesmol (200 μM) also inhibited HT29 cell migration toward type IV collagen and fibronectinline with inhibition of 76% and 63%, respectively [52]. Abnormal angiogenesis was involved in diverse diseases, such as tumor and diabetic retinopathy. Recently, β-eudesmol showed an inhibitory effect on angiogenesis partly via the arrest of the ERK signaling pathway, suggesting that β-eudesmol could be considered as the drug candidate for treatment of angiogenic diseases. In detail, β-eudesmol had an inhibitory effect on the multiplication of porcine brain microvascular endothelial cells and human umbilical vein endothelial cells (HUVEC). Additionally, β-eudesmol could inhibit the migration of HUVEC, and at the high concentration β-eudesmol could inhibit the ERK1/2 phosphorylation and suppress angiogenesis in subcutaneously implanted Matrigel plugs in mice and in adjuvant-induced granuloma in mice [58]. Another study showed that β-eudesmol had an inhibitory effect on angiogenesis via inhibiting CREB activation in growth factor signaling pathway, herein demonstrating β-eudesmol as an inhibitory compound of tumor growth. β-Eudesmol at the concentration ranging from 50 to 100 μM had an inhibitory effect on the proliferation of HUVEC stimulated with vascular endothelial growth factor (VEGF, 30 ng/ml) and basic fibroblast growth factor (bFGF, 30 ng/ml). In addition, β-eudesmol at the concentration of 100 μM also had a blocking effect on the phosphorylation of cAMP response element binding protein (CREB) triggered by VEGF 30 ng/ml in HUVEC. β-Eudesmol at the concentration ranging from 10 to 100 μM suppressed proliferation of HeLa, SGC-7901, and BEL-7402 tumor cells in a time-and dose-dependent manner. Moreover, treatment with β-eudesmol (2.5–5 mg/kg) markedly suppressed the growth of H22 and S180 mouse tumor growth in vivo [59]. Pheochromocytoma mainly refers to a neuroendocrine tumor of the adrenal glands. β-Eudesmol could induce neurite extension in rat pheochromocytoma cells (PC-12), accompanying with inhibition of [^3^H] thymidine incorporation. Meanwhile, β-eudesmol also stimulated the improvement of [Ca^2+^]. Moreover, β-eudesmol concentration-dependently led to an accumulation of inositol phosphates, and β-eudesmol could increase the p-MAPK time-dependently [60]. Recently, β-eudesmol was reported to modify the sensitivity of diabetic mice to depolarizing blockers so as to increase the susceptibility to these compounds. By investigating phrenic nerve-diaphragm muscles in normal and alloxan-diabetic mice, β-eudesmol exhibited the potentiating/promoting effect on neuromuscular blockade. Pretreatment with β-eudesmol enhanced the blocking action of succinylcholine to a greater degree in diabetic muscles than in normal ones. 30-min pretreatment in normal muscles could make the effect saturated,while 60-min pretreatment in diabetic muscles could make further potentiation [61]. It was reported that β-eudesmol could block the nicotinic acetylcholine receptor (nAChR) channel in both open and closed conformations, resulting in potentiating the neuromuscular blockade induced by succinylcholine (SuCh). The blocking effect of SuCh (0.1–10 μM) with β-eudesmol on nAChR channel activity was investigated by using the cell-attached patch clamp technique. Pretreatment with β-eudesmol (20 μM) had no effect on resting membrane potential and ACh-activated channel activities. β-Eudesmol decreased SuCh (above 0.3 μM)-induced prolongation of channel open time and reduced the frequency of channel opening in the presence of SuCh (above 3 μM) in ACh-activated channel currents regulated by SuCh [62]. β-Eudesmol could potentiate the effect of phenylene-polymethylene-bis-ammonium (PMBA) derivatives on neuromuscular blockades in alloxan-diabetic mice [63]. Current study manifested that the intragastric injection of β-eudesmol to rats could inhibit efferent adrenal sympathetic nerve activity (ASNA). The knock-out of TRPA1 could block the inhibitory effect of β-eudesmol on ASNA, and subdiaphragmatic vagotomy could promote the suppression of β-eudesmol on ASNA, suggesting that β-eudesmol could modulate ASNA through TRPA1 and afferent vagus nerve [7]. It was reported that β-eudesmol had the ability of modifying the chemical composition of the workers cuticle, to impair nestmate recognition, initiate alarm behavior and result in nestmate aggression [64]. Recent study showed that β-eudesmol pre-treatment on mouse diaphragm muscles for 30–60 min could promote [Ca^2+^] and twitch tension induced by succinylcholine as nicotinic acetylcholine receptor channel [65].

### Atractylodin

Atractylodin (Atr), a polyethylene alkyne, could be extracted from AL, exerting the anti-inflammatory activities. The melting point of Atr is 52 °C. A present study showed that atractylodin (Atr) could inhibit LPS-induced inflammatory responses. Namely, Atr could suppress myeloperoxidase (MPO) activity, the wet-to-dry weight ratio of the lungs, protein leakage, infiltration of inflammatory cells, TNF-α, IL-6, IL-1β and monocyte chemoattractant protein (MCP)-1 secretion. The mechanism was that Atr could down-regulate nucleotide-binding domain- (NOD-) like receptor protein 3 (NLRP3) inflammasome and TLR4 activation [66]. Another study reported that Atr exhibited anti-inflammatory effects and ameliorated concurring dysmotility in constipation-prominent (CP) and diarrheaprominent (DP) rats. The detailed mechanism was associated with the reduction of the plasma pro-inflammatory cytokines such as TNF-α, IL-1β, and IL-6 [67].

### Stigmasterol

Stigmasterol is an unsaturated phytosterol, and could extracted from soybean, calabar bean, rape seed, American Ginseng, AL and etc. The melting point of stigmasterol is 160–164 °C. Stigmasterol showed diverse pharmacological effects, such as anti-inflammotory, anti-nociceptive and anti-diabetic activities. Recent study exerted that stigmasterol could inhibit OVA-induced airway inflammatory damage in guinea pigs through intraperitoneal injection. More precisely, stigmasterol could suppress eosinophils, lymphocytes, and monocytes proliferation and inhibit peribronchiolar, perivascular, and alveolar infiltration of inflammatory cells. Stigmasterol could also inhibit vascular cell adhesion molecule-1 (VCAM-1) and OVA-specific immunoglobulin E (OVA sIgE) expression. VCAM-1 could medicate the adhesion of lymphocytes, monocytes, eosinophils, and basophils to vascular endothelium after the endothelial cells are stimulated by cytokines [68]. Recent study showed stigmasterol could improve memory and behavioral impairments in vanadium-induced neurotoxicity. More precisely, stigmasterol could reduce escape latency and prolong swimming time in Morris water maze. Also, stigmasterol could promote activities of antioxidant enzymes, and inhibit oxidative stress markers as well as lipid peroxidation in mice hippocampal homogenates [69]. A recent study showed that stigmasterol could protect mice from LPS-induced fever response. Further study exerted that stigmasterol relived organ damage and dearth rate [70]. Recently, stigmasterol exerted the synergistic antibiotic effect as adjuvant of ampicillin against both Gram positive and Gram-negative bacteria from clinical isolates. The inhibitory effect of ampicillin and stigmasterol alone is extremely ineffective, but the combination of stigmasterol-ampicillin significantly suppress colony counts, yielding 98.7% [71]. *Trypanosoma congolense* could induce the disease nagan in diverse animals. Stigmasterol showed antitrypanosomal activity against *Trypanosoma congolense* infection through reducing sialidase [72]. Moreover, a present study exhibited that stigmasterol displayed a mosquito larvicidal activity through neurotoxicity [73]. A present study displayed that stigmasterol showed anti-nociceptive effect on male albino Swiss mice model according to the results from acetic-acid writhing test, surgical incision, partial sciatic nerve ligation and complete Freund’s adjuvant. Further study exerted that naloxone could not block the anti-nociceptive effect of stigmasterol [74]. Recent study indicated that stigmasterol exhibited anti-diabetic activity in vitro and in vivo. Stigmasterol could increase GLUT4 translocation and expression. In the animal model, stigmasterol could reduce insulin resistance and oral glucose tolerance with the decrease of fasting blood-glucose levels and blood lipid indexes [75]. Recently, stigmasterol and β-sitosterol could suppress colitis in dextran sulfate sodium (DSS)-induced colitis in C57BL/6J male mice fed a high fat Western-style diet. β-Sitosterol and stigmasterol critically suppressed colon shortening, decreased fecal hemoglobin content, and inhibited the severity of colitis in the middle as well as distal colon through the inactivation of NF-κB. Particularly, stigmasterol could reduce COX-2 and CSF-1 [76].

### β-Sitosterol

β-Sitosterol is a phytosterol, and could be extracted from *Nigella sativa, Serenoa repens*, *Cucurbita pepo, Pygeum africanum*, AL and etc. The melting point of β-sitosterol is 136–140 °C. β-Sitosterol displayed cytotoxic effect on cancer cells and an anti-inflammatory activity. Multiple myeloma refers to a cancer of plasma cells. Recently, it was reported that β-sitosterol could display cytotoxicity on multiple myeloma U266 cells through apoptosis. Further study showed that β-sitosterol could increase production, activate AMP-activated protein kinase (AMPK) acetyl-CoA carboxylase (ACC) and JNK pathways, with a decrease of p-AKT, S6K and vascular VEGF [77]. Gastric cancer refers to the cancer starting from stomach. Recently, β-sitosterol exerted the anti-cancer effect on AGS human gastric adenocarcinoma cells through apoptosis. Further study showed that β-sitosterol could increase phosphatase and tensin homolog (PTEN) and p-AMPK expression, and decrease Hsp90 protein. Moreover, β-sitosterol could inhibit tumor weight and volume according in AGS xenograft animal study [78]. Recent study showed that β-sitosterol exerted the anti-inflammatory activity on rat model according to the rat paw edema test and the rat pleurisy assay [79]. A recent study manifested that β-sitosterol could ameliorate high fat diet induced dyslipidemia and hepatotoxicity in Swiss albino mice [80].

## Conclusion

In recent time, herbs and extractions from TCM have been widely considered as the complementary and alternative medicine (CAM) for various diseases treatment [81, 82]. AL has been used as TCM for thousands of years, and current studies found that AL and some of its pure compounds exerted diverse biological activities. It is interesting to find that most of major components could exert cytotoxic effects on cancer cells, especially melanoma (Table 2). However, the animal studies are insufficient. Also, the effects of these drugs on normal cells and healthy animals are lacking. Meanwhile, it is worthwhile to explore the pharmacological activities of minor components identified in AL. Nevertheless, further studies to identify other pure compounds for drug development and novel pharmacological activities of AL are still necessary to facilitate research and development.

**Table 2:** The anti-cancer effects of major components of AL

Chemical name	Tumor suppressive effect	Potential targets	Function study	References
Atractylenolide I	Anti-bladder cancer effect	↓PI3K/Akt/mTOR	In vitro and In vivo	[17]
	Anti-leukemia effect	↑CD14/CD68	In vitro	[23]
	Anti-melanoma effect	↓p-JAK2/p-STAT3	In vitro	[25]
Atractylenolide II	Anti-melanoma effect	↓p-STAT3/p-Src	In vitro and in vivo	[33]
	Anti-gastric cancer effect	↓p-Akt/p-ERK	In vitro	[34]
Hinesol	Anti-leukemia effect	↓c-Jun	In vitro	[50]
β-Eudesmol	Anti-bile duct cancer effect	↓NQO1	In vitro and in vivo	[53, 54]
β-Sitosterol	Anti-melanoma effect	↓JNK	In vitro	[77]
	Anti-gastric cancer effect	↑PTEN/p-AMPK	In vitro and in vivo	[78]

## Abbreviations

ACC: acetyl-CoA carboxylase
AL: *Atractylodes lancea* Thunb. DC
AMPK: AMP-activated protein kinase
ASNA: adrenal sympathetic nerve activity
ATL-I: atractylenolide I
ATL-II: atractylenolide II
ATL-III: atractylenolide III
Atr: atractylodin
bFGF: basic fibroblast growth factor
[Ca^2+^]: Ca^2+^ concentration
CAM: complementary and alternative medicine
CCA: cholangiocarcinoma
CP: constipation-prominent
CREB: cAMP response element binding protein
COX-2: cyclooxygenase-2
DP: diarrheaprominent
DSS: dextran sulfate sodium
EGF: epidermal growth factor
G-CSF: granulocyte-colony stimulating factor
HUVEC: human umbilical vein endothelial cells
5-HT: 5-hydroxytryptamine
IAV: influenza A virus
IFN-β: tumor necrosis factor receptor-associated factor 6 interferon-β
IL-8: interleukin-8
JNK: c-Jun N-terminal kinase
KPS: Karnofsky performance status
LPS: lipopolysaccharide
MAPK: mitogen-activated protein kinase
MCP: monocyte chemoattractant protein
MPO: myeloperoxidase
nAChR: nicotinic acetylcholine receptor
NO: nitric oxide
NOD: nucleotide-binding domain
NQO1: NAD(P)H-quinone oxidoreductase 1
OV: opisthorchis viverrini
OVA: ovalbumin
OVA sIgE: OVA-specific immunoglobulin E
PE: petroleum ether
PET-CT: positron emission tomography-computed tomography
PGE2: prostaglandin E2
PIF: proteolysis-inducing factor
PMACI: phorbol 12-myristate 13-acetate and calcium ionophore A23187
PMBA: phenylene-polymethylene-bis-ammonium
PTEN: phosphatase and tensin homolog
SuCh: succinylcholine
TFF2: trefoil factor2
TLR4: toll like receptor 4
TNF-α: tumor necrosis factor-α
UGT: uridine 5′-iphospho-glucuronosyltransferases
VCAM-1: vascular cell adhesion molecule-1
VEGF: vascular endothelial growth factor
